# Individual Differences and Features of Self-reported Memory Lapses as Risk Factors for Alzheimer Disease Among Adults Aged 50 Years and Older: Protocol for a Coordinated Analysis Across Two Longitudinal Data Sets

**DOI:** 10.2196/25233

**Published:** 2021-05-14

**Authors:** Jacqueline Mogle, Nikki L Hill, Jennifer R Turner

**Affiliations:** 1 Edna Bennett Pierce Prevention Research Center College of Health and Human Development Pennsylvania State University University Park, PA United States; 2 College of Nursing Pennsylvania State University University Park, PA United States

**Keywords:** subjective memory, individual differences, Alzheimer disease, daily assessment, multilevel modeling, coordinated analysis, mobile phone

## Abstract

**Background:**

Increasing evidence has promoted the clinical utility of self-reported memory problems for detecting early impairment associated with Alzheimer disease (AD). However, previous studies investigating memory problems often conflated the *types* of problems (ie, retrospective and prospective) with their *features* (ie, frequency and consequences). This bias limits the specificity of traditional measures of memory problems and minimizes their ability to detect differential trajectories associated with cognitive decline. In this study, we use a novel measure of self-reported memory problems that uses daily reports of memory lapses to disentangle *types* from *features* for analyzing the impact of each dimension in two longitudinal data sets. Furthermore, this study explores the individual difference factors of age and gender as potential moderators of the relationships between self-reported memory lapses and objective cognitive decline.

**Objective:**

The aim of this study is to describe the protocol for a secondary data analysis project that explores the relationship between experiences of daily memory lapses and their associations with cognitive decline in middle-aged and older adults.

**Methods:**

This study uses multilevel, coordinated analyses across two measurement burst data sets to examine the links between features and consequences of memory lapses (retrospective and prospective) and their association with objective cognitive decline. This study’s sample (N=392; aged 50-85 years; n=254, 64.8% women) is drawn from two ongoing, nationally funded research studies: The Effects of Stress on Cognitive Aging, Physiology, and Emotion study and the Einstein Aging Study. Both studies assess the daily experience of memory lapses, including the type as well as the emotional and functional outcomes, and objective measures of cognition, such as processing speed and episodic memory. We will use multilevel modeling to test our conceptual model demonstrating that differences in frequency and types of memory lapses show differential trends in their relationships with cognitive decline and that these relationships vary by the age and gender of participants.

**Results:**

This project was funded in August 2019. The approval for secondary data analysis was given by the institutional review board in February 2020. Data analysis for this project has not yet started.

**Conclusions:**

The early and accurate identification of individuals most at risk for cognitive decline is of paramount importance. Previous research exploring self-reported memory problems and AD is promising; however, limitations in measurement may explain previous reports of inconsistences. This study addresses these concerns by examining daily reports of memory lapses, how these vary by age and gender, and their relationship with objective cognitive performance. Overall, this study aims to identify the key features of daily memory lapses and the differential trajectories that best predict cognitive decline to help inform future AD risk screening tools.

**International Registered Report Identifier (IRRID):**

DERR1-10.2196/25233

## Introduction

### Background

Alzheimer disease (AD) is insidious in its onset, with clinically detectable cognitive decline only emerging late in the trajectory [1,2]. Once an individual reaches a diagnostic threshold of cognitive impairment, the functional ability is already negatively affected and a critical period for intervention has been missed [3,4]. Therefore, the period during which cognitive testing is within normal limits but subtle cognitive changes are noticed by older adults, particularly in complex real-world environments, is a crucial target for the prevention or delay of AD onset in individuals at highest risk [2,5]. Self-reports of memory decline, particularly episodic memory, are the earliest and most central deficit of AD [6,7], appearing up to 15 years before objective cognitive deficits [1], and are of high clinical relevance due to the associated functional consequences [8]. Furthermore, report of a cognitive concern is a required criterion for diagnosing mild cognitive impairment (MCI), and problems with memory are specifically associated with the highest risk of progression from MCI to AD [9].

### Self-reported Memory Problems and AD Risk

**Figure 1:**
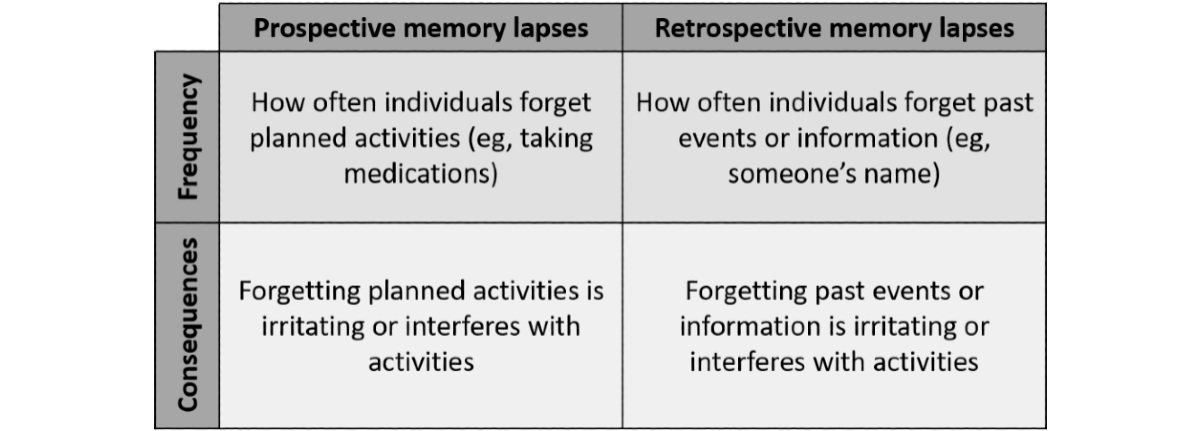
Features of memory lapses by the type of memory problem.

A growing body of evidence demonstrates the importance of memory problem reports in the risk profile for cognitive decline and AD. Cognitively intact older adults who report memory problems are up to four times more likely to develop AD over time than their peers who do not endorse problems [10-15]. Although several longitudinal studies demonstrate an increased risk of AD among older adults with self-reported memory problems, associations between objective cognition and reported memory problems are inconsistent [16,17]. Individuals who report memory problems are a decidedly heterogeneous group; only a subgroup is actually experiencing very early, subtle changes in their objective cognitive functioning that may indicate AD [18]. To distinguish insidious AD symptomology from memory problems because of other causes, it is important to better characterize the earliest cognitive symptoms, specifically examining the relationships between specific features of different types of reported memory problems (eg, prospective and retrospective memory lapses; Figure 1) and objective cognitive outcomes, and to further consider how age and gender (potential contributors to self-schemas that may influence reporting) affect these relationships.

### Daily Self-reported Memory Problems

Traditional measures of memory problems (eg, “Do you have problems with your memory?”) are inherently prone to response bias as they often require respondents to report experiences or changes with their memory functioning over long time periods or record a momentary snapshot of global functioning [19]. Historically, these measures do not distinguish between two important *features* of memory problems: the *occurrence* (ie, *frequency*) of problems and their *consequences*, namely, emotional (eg, worry, sadness) or functional (eg, reduction in activities). First, it is critical to understand how often individuals have memory problems. The frequency of memory problems, particularly in daily life, is related to the objective measures of memory and is higher in individuals with amnestic MCI than in healthy controls [20,21]. In addition, better cardiovascular fitness is associated with fewer forgetting episodes through the hippocampal volume, thereby suggesting a role for brain health in the experience of forgetting [22]. However, the frequency of memory problems is difficult to estimate accurately, given the extended time frames for reporting. Current measures tend to include consequences in questions about the frequency of memory problems, conflating the two and potentially reducing the predictive validity of reported memory problems on objective cognitive outcomes and the risk for AD. When self-reports of memory problems co-occur with general concerns about memory [23] or lower performance in independent activities of daily living (IADL) [15,24,25], the risk of future cognitive decline and AD is higher than with memory problem reports alone. Self-reported problems with remembering appointments and managing finances are better predictors of cognitive decline than other types of cognitive problems, such as paying attention to a television program [25,26]. Importantly, memory lapses associated with higher levels of consequences may also indicate that an individual is beginning to experience more severe memory problems, that is, memory problems associated with greater functional impairment. Memory problems *of consequence* may be better early indicators of cognitive decline. Assessing memory problems in naturalistic settings using a method that can uncouple occurrence from exposure would allow earlier detection of impaired memory. However, traditional memory lapse measures do not dissociate the consequences of memory problems from the frequency of their occurrence.

In addition to failing to separate frequencies and consequences, few measures assess different *types* of memory problems. Lab-based work suggests that self-reports of retrospective memory problems, or forgetting events from the past, may reflect decrements in the episodic memory, whereas self-reports of prospective memory problems, or forgetting future intentions, may be more closely related to executive functioning deficits [27,28]. Furthermore, prospective memory is associated with several factors key to successful aging, including IADL performance [29,30], quality of life [31], and medication adherence [32]. Although some multi-item assessments include both retrospective and prospective memory problems (eg, memory functioning questionnaire [33] and prospective and retrospective memory questionnaire [34]), these are rarely implemented in large population-based studies examining cognitive decline [35,36]. Given the evidence supporting the differential relationship of memory problem type (ie, retrospective vs prospective) with a variety of cognitive [27,28] and functional outcomes [30,37], it is important to examine how the frequency and consequences of different types of memory problems affect long-term cognitive performance.

### Influences of Age and Gender on Self-reported Memory Problems

Another important factor influencing the association between self-reported memory problems and objective cognitive decline is variation because of individual differences. Age and gender are primary nonmodifiable risk factors for AD, but neither of these have been extensively examined for their potential impact on the expression of reported memory problems or their cognitive outcomes [38]. Most research exploring self-reported memory problems is focused exclusively on older adults (ie, ≥65 years) because of the increase in AD risk with age. However, AD neuropathology is known to accumulate over years or even decades before diagnosis [39]. Cognitively intact middle-aged adults who report experiencing memory lapses exhibit structural brain differences consistent with AD as well as poorer memory performance than their peers [40,41]. Age may also play an important role in reports of memory problems because cognitive demands vary at different life stages (eg, before and after retirement), and different meaning is attributed to memory problems during middle age compared with later in life [42]. Depending on the operationalization of memory problems (eg, frequency and consequences), some studies have found no age effects [43,44], others have found an increase in self-reported memory lapses with age [45,46], and other have found differing nonlinear relationships across middle- and older age [47,48]. Thus, examining the features (ie, frequency and consequences) of different types (ie, retrospective and prospective) of memory problems is key to explicating these aging-related trends.

Although some memory problem features may increase with age, it is unclear how specific memory problems change over time or whether there are differential consequences from middle age to the oldest ages [12]. Older adults may be prone to reporting more serious consequences to memory problems considering increases in frequency over time [49]. Changes in memory performance may elicit anxiety regarding possible cognitive decline or AD or cause a loss of confidence in the ability to perform household activities or IADL [50]. In contrast, it is also possible that the consequences of memory problems decrease with aging, as individuals adapt to changing memory performance and develop appropriate compensation strategies [51]. Changes in memory are expected events among older adults [52]; therefore, they may be less emotionally and functionally burdened by their forgetting than their younger counterparts.

Regarding gender differences, women have a different risk profile for cognitive decline compared with men, including up to twice the risk of developing AD over their lifetime [53] and a more precipitous decline after the onset of a clinically identifiable deficit [54,55]. Gender differences in the rates of self-reported memory lapses are largely unknown; one early study found a higher prevalence in women [56] and another study found a higher prevalence in men [45]. Recent evidence suggests that women may report a greater frequency of memory lapses than men with similar objective cognitive performance [57]. This result may be attributed to a greater overall somatic symptom reporting by women [58]; however, it is critical to distinguish differential symptom reporting in women from illness or disease risk. Major depressive disorder, for example, is more common in women, but profiles of depressive symptom reporting demonstrate no gender differences [59]. The potential differences in the frequency and consequences of reported memory problems by gender are unknown, as are their associations with cognitive decline and AD.

### Conceptual Framework

**Figure 2:**
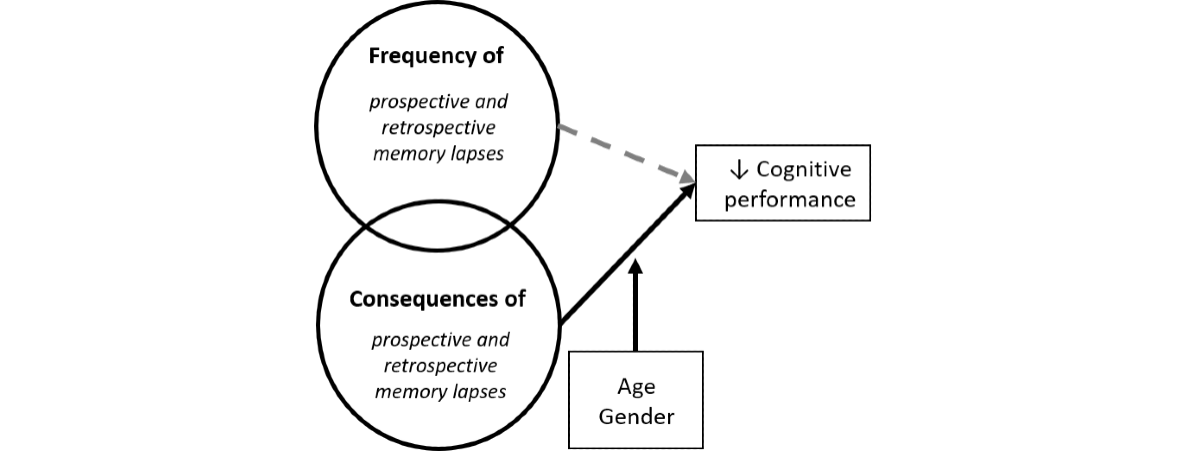
Conceptual model of this study’s aims.

The conceptual model guiding this study (Figure 2) is based on the identified need to disentangle two different aspects of memory problems: *occurrence* (ie, frequency) from functional and emotional *impacts* (ie, consequences), and gauge their unique contributions to the prediction of objective cognitive decline. This conceptual model additionally includes the key individual difference measures of age and gender, which may affect the expression and strength of the relationship between memory problems and cognitive performance. We propose to separate these features of memory problems by measuring memory lapses that occur on a daily basis using intensive, diary assessments that allow participants to provide more details about the memory lapses as they occur in their natural environment.

### Study Aims

The overall aims of this study are to examine how features of different types of memory lapses relate to objective cognitive performance and whether these associations depend on age or gender. Using a construct-level replication framework across two longitudinal data sets, we will examine the following aims:

- Aim 1: To test whether the frequency or consequences of different types of daily memory lapses (retrospective and prospective) predict decline in cognitive performance. We hypothesize that the consequences of memory lapses, rather than frequency, will better predict future cognitive decline.
- Aim 2: To identify age and gender differences in frequency and consequences of different types of daily memory lapses. We hypothesize that older adults will report more frequent memory lapses but rate these lapses as lower in consequences relative to middle-aged adults. For gender, we hypothesize that women will report more frequent memory lapses and rate memory lapses as having greater consequences compared with men.
- Aim 3: To test whether age or gender moderates the predictive utility of frequency or consequences of different types of daily memory lapses on cognitive decline. We hypothesize that age and gender will moderate the relationship between memory lapses and objective cognitive decline such that cognitive decline will be greatest for women and older adults reporting memory lapses with the highest level of consequences.

## Methods

### Overview

We will use multilevel modeling (MLM) in coordinated analyses in two measurement burst data sets funded by the National Institute on Aging (NIA): the Effects of Stress on Cognitive Aging, Physiology, and Emotion (ESCAPE) study [60] and the Einstein Aging Study (EAS) [61]. These data sets include intensive measurement components that are repeated multiple times across longer time frames, providing both daily data to capture the features of different types of daily memory lapses and long-term cognitive change on objective assessments (Multimedia Appendix 1 presents an overview of data collection protocols in ESCAPE and EAS). Critically, the application of MLM to intensive measurement designs such as ESCAPE and EAS permits the evaluation of within-person (ie, differences at the day level) and between-person variations (eg, individual differences), with the key addition of modeling *developmental change*. As participants in these ongoing studies are evaluated repeatedly across years, MLM can further address differences in developmental trajectories, including identifying the profiles of those individuals most at risk for developing cognitive impairment.

### Sample Characteristics

**Figure 3:**
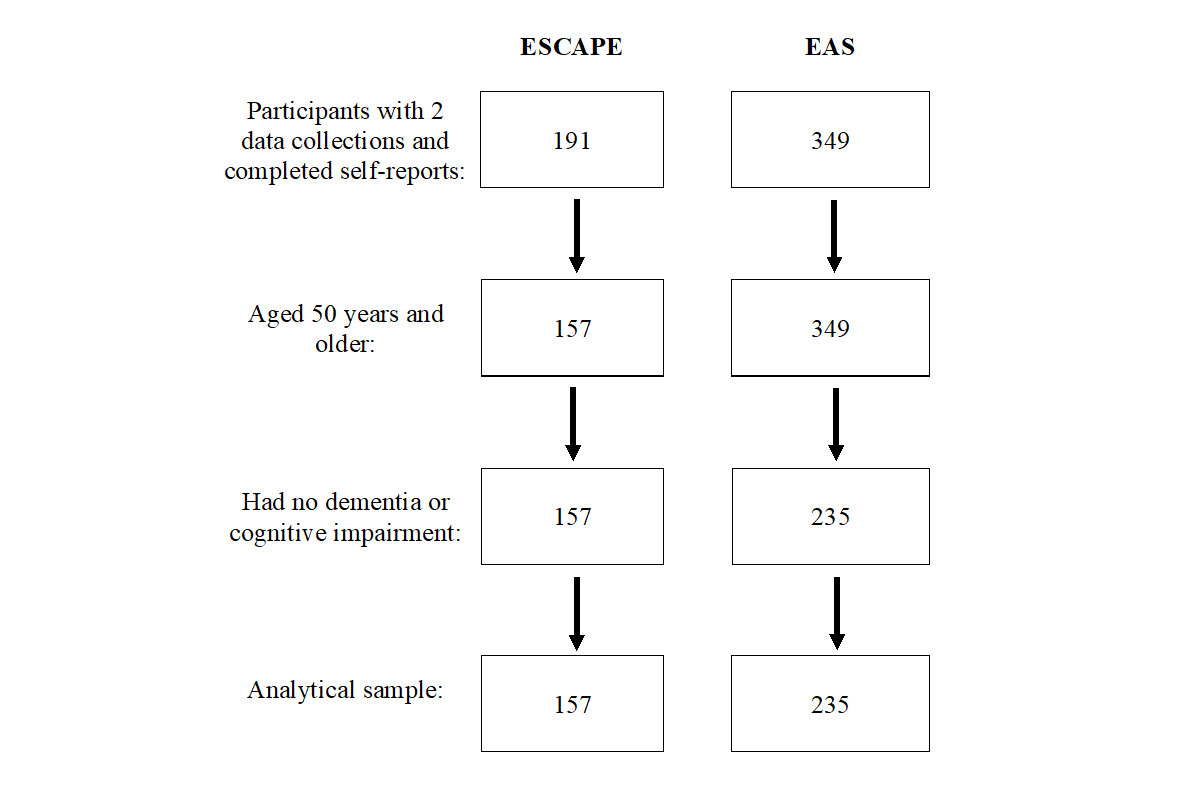
Sample size from the Effects of Stress on Cognitive Aging, Physiology, and Emotion study and Einstein Aging Study based on the inclusion criteria. EAS: Einstein Aging Study; ESCAPE: Effects of Stress on Cognitive Aging, Physiology, and Emotion.

Participants in this study must meet the following criteria for inclusion: age 50 years or older, no clinically significant objective memory impairment (ie, MCI or dementia) at baseline, and completion of at least two burst assessments for the longitudinal analysis (Figure 3 shows the flowchart of the current analytical sample in this study). The samples and design characteristics of the data sets are listed in Table 1. The ESCAPE and EAS data sets are recruited through systematic random sampling using a sampling frame from registered voter lists from Bronx, New York, and collected at an academic institution [60].

**Table 1:** Sample and design characteristics.

Characteristics			ESCAPE^a^		EAS^b^
**Sample description**					
	Total sample, n	157		235	
	Age (years), range	50-65		60-85	
	Gender: women, n (%)	99 (63.1)		155 (65.9)	
	Race: Black, n (%)	44 (28.0)		87 (37.0)	
**Study design**					
	Daily assessments: possible days, n	14		14	
	Possible bursts, n	4		4	
	Possible number of occasions. n	8792		13,160	

^a^ESCAPE: Effects of Stress on Cognitive Aging, Physiology, and Emotion.

^b^EAS: Einstein Aging Study.

### Designs and Procedures of Selected Data Sets

The included data sets are uniquely suited to our planned analyses because of the use of a measurement burst design and inclusion a measure of daily memory lapses that can be separated into frequency, emotional consequences, and functional consequences by retrospective and prospective memory lapses. Participants in both studies completed electronic daily diaries using a study-provided smartphone that guided participants through data collection and provided a date and time stamp for each observation. These time stamps were critical to assuring that diaries were completed as instructed rather than at the end of the diary period (ie, backward filling) [62,63]. Each study also included extensive cognitive testing (traditional and ambulatory) and a questionnaire battery for physical health and psychological well-being. The primary differences among the study designs were the selection of lab-based assessments for cognition (Textbox 1), psychological well-being, and physical health. ESCAPE finalized the collection in 2019, and data collection in the EAS is ongoing.

The daily diary design of these projects specifically supports our measurement approach for daily memory lapses. Participants report on their experiences with memory lapses at the end of the day report, and for any memory lapses experienced, they provide additional details on the impact of that lapse. Reporting at the end of the day, rather than over longer time windows, reduces a recall bias in reporting and allows a greater recollection of experiences and their impact.

**Objective cognition measures by study.**

Episodic memory

- Effects of Stress on Cognitive Aging, Physiology, and Emotion (ESCAPE) study
  Paired Associates
  Spatial Location Memory
  Auditory Verbal Learning Test
- Einstein Aging Study (EAS)
  Logical Memory
  Craft Story
  Benson Complex Figure

Working memory

- ESCAPE
  Operation Span
  Backward Letter Span
- EAS
  Backward Number Span

Other cognition

- ESCAPE
  Shipley Vocabulary
  Ravens Progressive Matrices
- EAS
  Trails A/B
  Digit Symbol
  Wechsler Adult Intelligence Scale Vocabulary
  Multilingual Naming Test
  WAIS-III Block Design

### Ethics Approval and Consent to Participate

Data collection in the EAS and ESCAPE data sets was approved by the institutional review board at the Albert Einstein College of Medicine, and participants provided written informed consent for participation. This study was approved by the Pennsylvania State University Institutional Board (STUDY00012793 [ESCAPE] and STUDY00017272 [EAS]). Informed consent for this project was waived by the institutional review board because of the exclusive use of secondary data sets.

### Measures

#### Memory Lapses

Both data sets include a measure of daily memory lapses. Retrospective memory lapses are represented by lapses for names, words, past events or information, and where something was placed. Prospective memory lapses are represented by lapses for medications, appointments, chores, and finishing something that was started. For both types of lapses, the participants are asked two follow-up questions. The first asks about emotional consequences (ie, “How much did this bother you?”) and the second asks about functional consequences (ie, “How much did this interfere with your activities?”). Both questions are rated on a visual analog scale ranging from 0 to 100.

#### Objective Cognition

Both data sets include a number of lab-based measures of objective cognition as well as novel ambulatory assessments of cognitive performance. The lab-based assessments include measures of episodic memory, working memory, executive functioning [64,65], vocabulary [66-68], spatial memory [69], and fluid intelligence [70].

**Figure 4:**
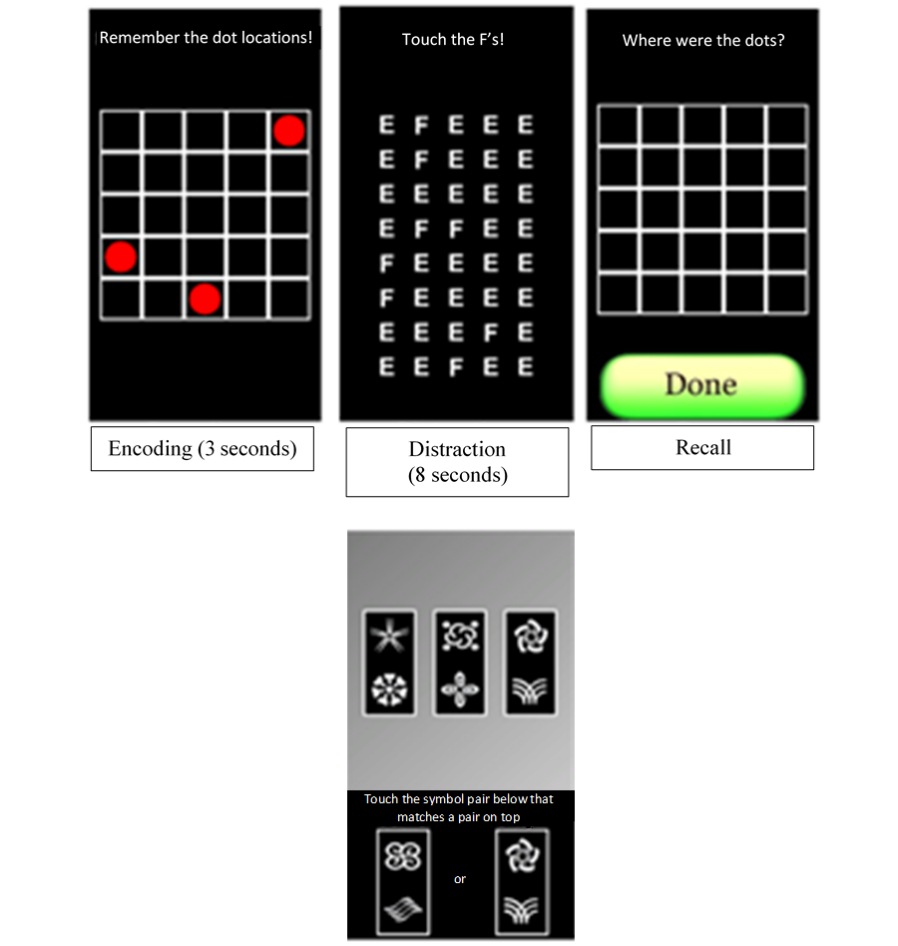
Ambulatory cognitive tests administered via smartphone. Top three images are the spatial memory test (in temporal order), and the bottom image is the processing speed test.

Ambulatory objective cognitive tests were administered remotely via smartphones (Figure 4). Participants completed several trials of these tests up to five times each day at a pseudorandomly determined time (spaced approximately 2-3 hours apart). At each assessment, the participants completed a processing speed and spatial memory test. The processing speed test uses the reaction time as an outcome. The spatial memory test uses an accuracy measure that quantifies the distance between the original and the participant’s indicated locations of the dots. The reliability of these assessments exceeds 0.95.

#### Covariates

Both studies include a detailed medical history questionnaire that can be used to identify medical conditions that may impact memory functioning, including endocrine disorders (eg, diabetes), cardiovascular diseases (eg, hypertension), and chronic inflammation (eg, arthritis), as well as the measures of depressive and anxiety symptoms to account for contributions of other psychological symptoms that are related to memory impairment.

### Availability of Data and Materials

The EAS and ESCAPE data sets are available from the Albert Einstein College of Medicine, but restrictions apply to the availability of these data. These data sets were used under license for this study, and so they are not publicly available. However, data are available under reasonable request from the authors and with permission of the principal investigators of EAS and ESCAPE as well as their affiliated organizations.

### Analysis Plan

We will use three-level MLM to examine which features of daily memory lapses (frequency or consequences of retrospective or prospective lapses) predict future cognitive decline (aim 1); the impact of age and gender on the frequency and consequences of different types of daily memory lapses (aim 2); and, finally, whether age or gender moderates the predictive utility of the frequency or consequences of daily memory lapses on cognitive decline (aim 3). MLM is appropriate when observations are nested, such as in this study’s data sets (days in bursts and bursts in persons) [71]. The MLM approach offers an advantage over other types of analytic models for repeated measures data (eg, repeated measures analysis of variance) for two reasons. First, MLM allows us to make use of all available data through maximum likelihood estimation methods rather than excluding individuals who fail to complete some surveys or who drop out of the study at later waves. Second, we can also test for individual differences among our relationships of interest by including random effects. We will explicitly test the underlying hypothesis that the consequences of memory problems that individuals experience in their everyday lives are the most informative for predicting future cognitive decline. Although the frequency of memory lapses is a necessary condition for the consequences of those lapses, we hypothesize that frequent memory lapses are not a sufficient predictor of cognitive decline over time. Analyses will be conducted by JM and JRT with assistance from NLH.

#### General Approach to Analysis

Across both data sets, we will begin by examining daily correlates of memory lapses to identify the potential confounds in the daily assessments that should be accounted for across analyses. Significant daily predictors of memory lapses will be incorporated into primary analytic models to control for other processes that influence the daily reporting of memory lapses. Potential daily covariates uncovered in the literature include daily stress [72] and physical activity [73].

#### Operationalization of Daily Memory Lapses

Measures of daily memory lapses across both data sets follow the same general structure with minor differences, allowing us to draw equivalent operationalizations of frequency and consequences across different memory lapse types. For the *frequency* of memory lapses, we will compute the total number of memory lapses over the daily diary period separately for each type of memory lapse (ie, prospective and retrospective). To measure *consequences*, we will use both the average and the maximum ratings across the daily diary period. In addition, we will separately examine the emotional and functional consequences for each of the different types of memory lapses.

#### Operationalization of Objective Cognitive Performance

Data from lab-based cognitive tests (eg, Shipley Vocabulary) will be scored using standardized methods. Moreover, data from ambulatory cognitive tests will be used to create scores reflecting each of the following indicators: average performance, upper quintile performance, lower quintile performance, and intraindividual variability. For all objective cognitive performance–based tests, we will first remove any anticipatory (<150 milliseconds) or delayed (>3 SDs above the mean) responses from distributions by examining response times for all trials [74]. After detrending the remaining data for practice effects as in our previous work [75], we will compute the average, lowest quintile, and highest quintile scores for each task for each individual at each burst. We will also compute residualized and raw intraindividual SDs as the indicators of variability in cognitive performance [76-78].

#### Approach to Coordinated Analysis

Coordinated analysis was selected for this study as it permits the efficient replication of results across data sets to generate stronger substantive conclusions. Moreover, it allows fitting individual models within each data set, testing of covariates, and comparison of the effects of interest across different samples and contexts [79-81]. Using equivalent parameterization ensures that models’ effects reflect the same underlying constructs across data sets and standardized estimates will promote the comparison of effects across data sets. We will conduct data set–specific follow-up analyses that focus on additional measures of cognitive performance to ensure the replicability of findings across different operationalizations. All cognitive data will be examined for practice effects before analysis.

##### Aim 1

Using MLM, we will first examine whether the frequency or consequences of different types of memory lapses covary with cognitive performance over time. This analysis addresses whether at assessments when an individual has a higher frequency of memory lapses (or reports higher levels of consequences), do they have poorer cognitive performance? Next, to test the prospective prediction hypothesis, we will use autoregressive MLM models to test the temporal relationships and determine whether changes in daily memory lapses from previous occasions predict future changes in cognitive performance over time. All models will examine the different features and types of memory lapses.

##### Aim 2

Potential contributor differences in the experience of daily memory lapses are the individual’s age and gender. We will explicitly examine the associations of age and gender with frequency and the consequences of different types of daily memory lapses. When the frequency of memory lapses of different types is the outcome, we will use multilevel Poisson regression models. Poisson regression is the most appropriate when the outcome is count data and when the counts are not normally distributed [82]. Both emotional and functional consequences were rated on a Likert scale and can be appropriately represented using a normal distribution [83].

##### Aim 3

For our third aim, we will include age and gender as the moderators of the predictive utility of frequency and consequences of daily memory lapses for predicting changes in cognitive performance. We will extend the analyses in aim 1 to include an interaction term between age at baseline (or gender) and frequency, as well as age at baseline (or gender) and consequences, to predict cognitive performance. We will then examine age and gender moderation for the frequency and consequences of the different types of memory lapses.

## Results

This project was funded by the NIA in August 2019 (see Multimedia Appendix 2 for reviews of current protocol) and was approved by the Pennsylvania State University Institutional Review Board (STUDY00012793 [ESCAPE] and STUDY00017272 [EAS]). Data analysis for this study has not yet begun, but data cleaning and preliminary analyses are expected to be completed by January 2021. All aim-specific analyses are expected to be completed by April 2023.

## Discussion

### Principal Findings

The early and accurate identification of individuals most at risk for cognitive decline, functional impairment, and increased risk of AD is critical for an early intervention. Older adults who report memory problems but do not have objective memory impairment are at a substantially higher risk of AD than those who do not report problems [1,84]. Despite previous work showing that reports of memory problems are sensitive to subtle cognitive decline [35,36], there are potential biases in traditional measures, such as perceptions of normative and nonnormative aging, which limit the clinical utility of these measures in the early detection of cognitive impairment. For example, younger adults are more likely to attribute forgetfulness to emotional difficulties or stress than older adults [42], whereas older adults are more likely to view memory problems as a normal part of aging and less concerning [44,52]. Alternatively, self-reported memory problems may be more salient to older adults [85], particularly given that the fear about AD is common among those who report memory problems [86] and/or have had a family experience with AD [86,87]. Differences between men and women follow a similar, contradictory pattern: some studies have found that women report more memory problems than men [35,53], whereas other studies found that reports of memory problems among men may be more predictive of functional impairment [38,45,88]. The lack of consistency between these results can likely be attributed to issues with traditional measures of self-reported memory problems that require individuals to recollect memory problems over months or years, aggregate these experiences, and report on them without distinguishing the frequency of experiences from the outcomes associated with the experience (eg, impacts on emotional and daily functioning) [16,89,90]. To increase the specificity of self-reported memory problems, we must refine our measures to account for the frequency and consequences of different types of memory problems.

This study addresses these previous limitations in memory problem assessment by using daily diary data collected in two NIA-funded longitudinal daily diary studies and a novel measure of daily memory lapses. This measure includes retrospective and prospective types of memory lapses and is collected daily over multiple bursts for both studies, which permits the investigation of frequency of occurrence and consequences without relying upon recollection and minimizes potential bias. By disentangling the components of self-reported memory lapses (ie, features and consequences) using daily measures, this project seeks to improve the specificity of memory lapse measures for predicting cognitive decline over time; measures that capture these additional characteristics of memory lapses may be more sensitive for detecting subtle cognitive decline earlier in the aging trajectory. A major strength of this study is the inclusion of potential modifiers of age and sex. Given the conflicting evidence regarding the relationship between self-reported memory lapses and objective measures of cognitive decline, the examination of these individual difference measures is necessary to identify the indicators of future cognitive risk and model varying developmental trajectories. Finally, the design of this study, using two large, representative data sets with up to 30,000 days of data, provides the opportunity for both coordinated analysis and direct construct-level replication.

### Conclusions

This study addresses the urgent need [84] to identify the indicators of future cognitive decline risk to inform the development of noninvasive AD risk screening tools and novel intervention targets. Identifying the components associated with the accurate prediction of reported memory problems is necessary to improve assessment specificity and the clinical utility of self-reported memory problems as a symptom. Daily measurements can capture different types of memory lapses that occur, their frequency, and their emotional and functional consequences. Examining these experiences earlier in the aging trajectory and considering individual differences (eg, gender) will establish more sensitive indicators of those adults most at risk, before the onset of functional decrements associated with cognitive decline. Early, easy-to-implement tools for the detection of AD risk are a key component of reducing individual and societal burden. These tools can provide the time needed for patients and families to plan for the future and mobilize resources, evidence to guide the enrichment of samples for future research, and the opportunity to develop tools for use in early intervention trials.

## Appendix

Multimedia Appendix 1 Overview of data collection protocols in the Effects of Stress on Cognitive Aging, Physiology, and Emotion study and Einstein Aging Study.

Multimedia Appendix 2 Redacted National Institute on Aging peer review of this protocol.

## Abbreviations

AD: Alzheimer disease
EAS: Einstein Aging Study
ESCAPE: Effects of Stress on Cognitive Aging, Physiology, and Emotion
IADL: independent activities of daily living
MCI: mild cognitive impairment
MLM: multilevel modeling
NIA: National Institute on Aging
