# Supplementary figures and images for: Individual Differences and Features of Self-reported Memory Lapses as Risk Factors for Alzheimer Disease Among Adults Aged 50 Years and Older: Protocol for a Coordinated Analysis Across Two Longitudinal Data Sets
Source: JMIR Res Protoc. 2021 May 14;10(5):e25233. doi: 10.2196/25233 (PMC8164128; doi:10.2196/25233)

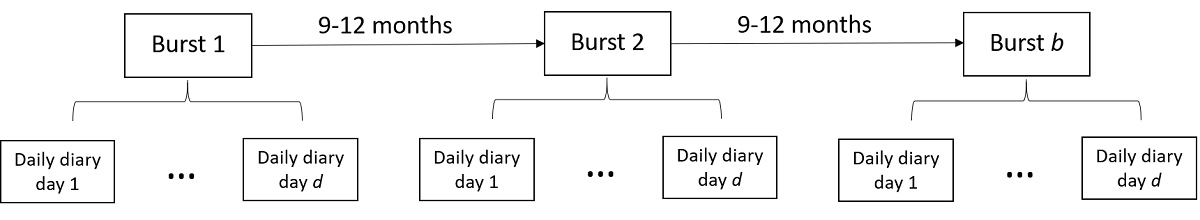

Supplement: Multimedia Appendix 1 [file resprot_v10i5e25233_app1.png]
