# Supplementary material for: Individual Differences and Features of Self-reported Memory Lapses as Risk Factors for Alzheimer Disease Among Adults Aged 50 Years and Older: Protocol for a Coordinated Analysis Across Two Longitudinal Data Sets
Source: JMIR Res Protoc. 2021 May 14;10(5):e25233. doi: 10.2196/25233 (PMC8164128; doi:10.2196/25233)
Supplement: Multimedia Appendix 2 [file resprot_v10i5e25233_app2.pdf]

SUMMARY STATEMENT

PROGRAM CONTACT:
[redacted]

(Privileged Communication)

Release Date: [redacted]
Revised Date:

Application Number: [redacted]

Principal Investigator

[redacted]

Applicant Organization: [redacted]

Review Group: ZRG1 PSE-Z (55)
Center for Scientific Review Special Emphasis Panel
PAR Panel: Epidemiology and Cohort Studies for Alzheimer's Disease, Related Dementias and Cognitive Resilience

Meeting Date: [redacted]
Council:
Requested Start:

RFA/PA: [redacted]
PCC:

Project Title: Improving assessment of subjective memory impairment for detecting Alzheimer's disease: A coordinated analysis in two measurement burst studies
SRG Action: [redacted]
Next Steps: Visit https://grants.nih.gov/grants/next_steps.htm
Human Subjects: E4-Human subjects involved - Exemption #4 designated
Animal Subjects: 10-No live vertebrate animals involved for competing appl.

[redacted]

EARLY STAGE INVESTIGATOR
NEW INVESTIGATOR

[redacted]

EARLY STAGE INVESTIGATOR
NEW INVESTIGATOR

RESUME AND SUMMARY OF DISCUSSION: This resubmission proposes to measure the Subjective Memory Impairment (SMI), the frequency and consequences of daily memory lapses as well as age and sex differences that could predict cognitive decline. Reviewers agreed that this application is significant as understanding the subjective memory complaint could have important implications for the early detection of cognitive decline. The application is supported by strong preliminary data on daily memory lapses and distinction between frequency, type and consequences of memory lapses. The use of the burst assessment of subjective memory that involves the daily measures of memory lapses over 2 weeks, and the incorporation of smartphone data were considered innovative and significant strengths. Other strengths of the application include the use of 2 well-characterized cohorts of aging, the plan to examine sex differences and effects moderation by sex, as well as a robust analytic plan. Still some concerns were expressed about the investigative team that may be missing an expert on cognitive decline and a modest publication record for the principal investigator. Also, there were concerns about the lack of details on the power calculations and potentially limited resources to analyze the smartphone data. Following discussion, most reviewers agreed that these weaknesses reduce the overall impact of this application in understanding subjective memory impairment to a moderate level.

DESCRIPTION (provided by applicant): The perception of memory problems without objectively identified memory deficit (subjective memory impairment, SMI) is associated with a substantially increased risk of future cognitive decline and Alzheimer's disease (AD). However, many individuals will endorse SMI but not experience a significant decline in cognition over time. This inconsistency is largely due to current limitations of SMI as an indicator: traditional SMI measures cannot discriminate who will eventually decline, limiting our ability to link the experience of SMI with AD risk. The science examining the SMI-AD link has relied on measures ask that ask individuals to reflect on their experiences with memory over long periods of time, and to remember what they've forgotten. This approach to measurement is susceptible to multiple sources of response bias, such as biased recall and a reliance on self-schemas about memory abilities (e.g., I'm bad at remembering names) rather than recent experiences with their memory (e.g., I forgot someone's name yesterday). Traditional measures also don't differentiate between problems with memory and the consequences of these problems (e.g., health effects of forgetting to take an important medication). In this early stage and new investigator application, we will address these problems by using daily assessments of memory problems (i.e., memory lapses) that occur in the real world to examine cognitive outcomes over time, rather than relying on traditional SMI measures. Daily assessments allow us to separately examine whether the occurrence of memory problems and their consequences serve as separable indicators of future cognitive decline. We will conduct coordinated analyses across two NIA-funded datasets that repeatedly assessed individuals every six to nine months over a period of three years. At each assessment, participants completed a daily diary that included questions about their memory lapses every day for up to 14 days, as well as ambulatory and lab-based tests of memory and other aspects of cognitive performance. Using these datasets, we will examine three aims: 1) test whether the consequences of daily memory lapses are a better predictor of cognitive decline compared with just the occurrence of problems; 2) test whether daily memory lapses and their consequences differ across individuals of different ages and between men and women; 3) test whether the consequences of memory problems are better predictors for individuals who are older, or for men compared with women. Determining what aspects of memory problems, such as their emotional or functional consequences, are better indicators of future cognitive decline would allow us to develop better tools to identify individuals who are at risk for poor cognitive outcomes, including AD. Additionally, if consequences are better predictors for some individuals (e.g., women) compared with others, we will be better prepared to tailor clinical tools to the individuals that are most likely to benefit from early identification.

PUBLIC HEALTH RELEVANCE: This project will examine characteristics of different types of daily memory problems, such as how often they occur and if they lead to negative consequences, in order to determine which characteristics are the best predictors of future cognitive decline. We will determine: 1) whether some characteristics of memory problems are better indicators of future cognitive decline; 2) whether the characteristics of memory problems depend on age or sex; and 3) if the characteristics of memory problems that predict cognitive decline depend on age or sex. This project will identify the key features of daily memory problems that best predict cognitive decline.

CRITIQUE 1

[redacted]

Overall Impact: Subjective Memory Impairment (SMI) is an important precursor to cognitive decline in many older individuals. The proposed project proposes to analyze previously collected SMI questionnaire, remotely collected cognitive testing and traditional cognitive testing data from two community based longitudinal studies of aging (ESCAPE and EAS) to improve measurement of SMI, understand sex differences and evaluate the predictive value for cognitive decline. The significance of understanding SMI and the innovative approach proposed for the study are clear strengths. However, there are major weaknesses in the approach, mainly lack to inadequate explanation of inclusion criteria, lack of enough preliminary data, and cursory power calculations that significantly detract from the study's overall impact. Additionally, the investigative team lacks a medical expert in cognitive decline who might added an added perspective to the analyses, and it isn't clear that the research environment would provide such expertise. It is also not clear that the investigators have the necessary resources present to analyze the smartphone data. It isn't clear from the resource sharing section that all the data from the current study will be shared. Overall, the application proposes to study a significant question using innovative techniques, however weaknesses in approach, investigators and environment are detract from the impact score.

1. Significance:

Strengths

- A better understanding of the meaning and consequences of subjective memory impairment (SMI) in elderly individuals may improve early detection of cognitive decline.
- There is strong evidence form the literature that SMI indicates increased risk of cognitive decline.
- Improved screening tools for SMI and remote measurement techniques could be useful for identifying individuals at risk for cognitive decline in the community.

Weaknesses

- In the absence of biological or longitudinal clinical diagnostic information regarding participants (e.g. a clinical gold standard) it is hard to know how useful a better understanding of SMI and improved SMI measurement techniques will be.

2. Investigator(s):

Strengths

- The principal investigator has extensive experience in the assessment of self-reported memory and in identifying the impact of daily memory lapses on affect.
- Strong track record of collaboration with the other investigators on development of ambulatory cognitive measurement tools.

Weaknesses

- A medical expert in diagnosis and biology of age-related cognitive decline would likely add value to the current investigative team.

3. Innovation:

Strengths

- Burst measurements of daily memory diaries is a novel approach to collecting subjective memory impairment data.
- Incorporation of smartphone-based questionnaires may improve data quality and reliability
- Will separate the effects of SMI frequency as compared to SMI consequences
- Use of smartphone based cognitive measures as an outcome is novel

Weaknesses

- It isn't clear how the current study is an advance over other studies from the same group such as [redacted] R01 on AD risk factors and SMI

4. Approach:

Strengths

- The study will build on two already established community aging studies ESCAPE and EAS, to which the investigative team contributed the inclusion of the currently proposed assessment measures.
- There is a well-developed, theoretical statistical model to relate the different measurements and other variables to the outcome measures.
- One of the aims will address sex as a biological variable that mediates presence and consequences of SMI.
- Examining reaction times in addition to other performance measures may increase sensitivity

Weaknesses

- Insufficient preliminary data on the smartphone based cognitive assessments are presented are shown to understand their feasibility and value to address the proposed aims.
- The inclusion criteria are confusing. If the data will be taken from the ESCAPE and EAS studies that are already underway, then how will the ADNI screening procedures be deployed in this study? Does Table 1 show the actual number of participants to be included or the potential number to be screened for inclusion in the current study?
- In part due to lack of preliminary data, the power calculations don't present enough detail to give confidence that the estimated effect sizes will be enough to test the hypotheses, particularly for the longitudinal hypotheses.
- The investigators acknowledge that if individuals don't report daily memory lapses, they can't execute the current study. They cite experience from a different study indicating that only 16% percent of individuals did not report daily memory lapses. Since the EAS and ESCAPE studies

are presumably already enrolled, it would make sense to verify that the necessary data exist in the planned dataset prior to proceeding.

5. Environment:
Strengths

- The investigators will benefit from a strong intellectual environment as members of different centers devoted to aging, geriatric nursing and methodology.

Weaknesses

- It isn't clear that the resources to use and analyze the smartphone and other computational data are present at the investigators' institution

Protections for Human Subjects:

Acceptable Risks and/or Adequate Protections

- The data will be acquired from two previously recruited and almost complete studies. Current study is mainly data analysis

Data and Safety Monitoring Plan (Applicable for Clinical Trials Only):

Not Applicable (No Clinical Trials)

Inclusion of Women, Minorities and Children:

- Sex/Gender: Distribution justified scientifically
- Race/Ethnicity: Distribution justified scientifically
- For NIH-Defined Phase III trials, Plans for valid design and analysis: Not Applicable
- Inclusion/Exclusion of Children under 18: Excluding ages <18; justified scientifically
- This is a study of SMI in elderly individuals

Vertebrate Animals:

Not Applicable (No Vertebrate Animals)

Biohazards:

Not Applicable (No Biohazards)

Resubmission:

- This is a resubmission that has been moderately responsive to previous critiques

Resource Sharing Plans:

Unacceptable

- It isn't clear whether all the relevant data from the study will be deposited in the Interuniversity Consortium for Political and Social Research

Authentication of Key Biological and/or Chemical Resources:

Not Applicable (No Relevant Resources)

Budget and Period of Support:

Recommend as Requested

CRITIQUE 2

[redacted]

Overall Impact: This is an innovative application investigating Subjective Memory Impairment (SMI) using daily memory lapse assessments via Smartphone, with focus on sex and age effects on different components of memory lapses (frequency, type and consequence) and their predictions of objective cognitive decline. The impact of this work will be a more sophisticated understanding of SMI and how to assess it to improve prediction of future cognitive decline. The significance is therefore high, but this is somewhat diminished by lack of AD biomarkers. The approach is strong, particularly the analytic modeling, though a minor concern is limited follow-up time. Overall, enthusiasm is high for the SMI approach and outstanding investigators, but somewhat diminished by limited available outcomes with which to test important questions about the detailed SMI assessments.

1. Significance:

Strengths

- Thoughtful, thorough and well-referenced background discussion on complexities of SMI and its measurement lends strength to the scientific premise, overall.
- Strong preliminary work from the study team presented on daily memory lapses and distinction between frequency and type, as well as assessing consequences of memory problems.
- Strong premise from the research team supporting use and analysis of ambulatory measurements in daily burst design to address important questions in cognitive aging.

Weaknesses

- Lack of AD biomarkers detracts from the stated scientific premise that this study addresses AD (and NAPA goals), specifically.

2. Investigator(s):

Strengths

- The study team is comprised of highly accomplished and recognized experts in the field of cognitive aging, SMI, and sophisticated analytic modeling.
- The principal investigator is experienced with daily ecological assessment techniques, and the daily memory lapse measurement implementation in the studies of focus. She has the skills and experience to lead the project.

Weaknesses

- None noted

3. Innovation:
Strengths

- Daily diary method and burst designs are a novel approach to understand measurement of SMI.
- The careful parsing of frequency, consequence and type of memory failures in an ecologically valid setting seeks the shift SMI field into more nuanced understanding of subjective cognition.

Weaknesses

- None noted

4. Approach:

Strengths

- Data sources are two representative longitudinal cohort studies with about 30% African-American participants.
- Analytic plan of multi-level modeling is strong and thoroughly detailed.
- Sex differences and effect moderation by sex is an Aim of the study.
- Robust approach is reflected by consideration of potential problems (e.g., high covariance among memory lapse variables; short follow-up time) and alternative approaches.

Weaknesses

- Prediction of cognitive decline on objective tests is the goal for 2 of 3 Aims, but the follow-up time is "up to four years", and it is not clear what the range or distribution of the follow-up time is. The power analyses are vague on this and do not specifies the outcome. An alternative approach is described to capitalize on intensive, repeated ambulatory cognitive measurements to estimate decline in shorter periods (e.g., 6-months), somewhat mitigating this concern.

5. Environment:
Strengths

- The environment at [redacted] is very strong and well suited to successfully support these Aims.

Weaknesses

- None noted

Protections for Human Subjects:

Acceptable Risks and/or Adequate Protections

Data and Safety Monitoring Plan (Applicable for Clinical Trials Only):

Not Applicable (No Clinical Trials)

Inclusion of Women, Minorities and Children:

- Sex/Gender: Distribution justified scientifically
- Race/Ethnicity: Distribution justified scientifically
- For NIH-Defined Phase III trials, Plans for valid design and analysis: Not Applicable
- Inclusion/Exclusion of Children under 18: Excluding ages <18; justified scientifically

Vertebrate Animals:

Not Applicable (No Vertebrate Animals)

Biohazards:

Not Applicable (No Biohazards)

Resubmission:

- The application was responsive to prior reviews.

Resource Sharing Plans:

Acceptable

Authentication of Key Biological and/or Chemical Resources:

Not Applicable (No Relevant Resources)

Budget and Period of Support:

Recommended budget modifications or possible overlap identified:

- The support of the numerous personnel described add up to quite a lot of effort for a secondary data analysis-only project.

CRITIQUE 3

[redacted]

Overall Impact: The purpose of this project is to do secondary data analysis of two longitudinal cohort studies to better understand the cognitive underpinnings of subjective memory complaint (SMC). SMCs appear useful to identifying incipient cognitive deficit but are limited by recall bias and a conflation of memory lapse events with the consequences of those events. This project will use "burst" (very frequent—daily over 2 weeks) assessment of SMCs to fractionate the SMC into these two compartments and see how these two components relate to objective cognitive decline; and differ by age and sex. Differences in the relationship to objective decline by age and sex will also be determined. Key score-driving strengths include the high clinical significance of SMCs; the innovative approach of fractionating them using burst measurement; and well described and rigorous methods. The only residual score-driving weakness is the principal investigator's publishing record as first author, which is still thin.

1. Significance:

Strengths

- The idea of fractionating subjective memory complaint (SMC) into component parts, specifically memory lapse incidents vs. consequences of memory lapses, is highly significant because SMCs appear to provide independent and useful information beyond objectively-measured memory impairment, in terms of prediction of subsequent objectively measured decline. The problem with SMCs has always been that it is unclear what (if any) objective events underlies SMCs, and therefore what their relation to brain disease might be. This project adds knowledge in that area.
- A previous concern that burst measurements are not scalable to large-scale cohorts seems to be a bit off-base. First, even if the sole purpose of burst measurement is to better understand traditionally-defined SMCs, they are valuable for this project. Second, now that EMA (ecologically momentary assessment) via cell phones has proven to be a viable technology, it does not seem like much of a stretch to collect this kind of burst measurements via push notifications on a large scale.

Weaknesses

- None noted.

2. Investigator(s):

Strengths

- The principal investigator is co-investigator on a large set of NIH-funded projects. She has highly promising expertise in neuropsychology of cognitive aging.
- The degree of participation of the consultants (previously noted as unclear) has now been clarified.

Weaknesses

- As noted previously, the principal investigator has only a small set of single-authored publications—apparently only 3. This is low for this career stage.

3. Innovation:

Strengths

- Using a “burst” approach as a means to tease apart individual memory lapse incidents versus consequences, and to reduce longer-term recall bias, is innovative.

Weaknesses

- None noted.

4. Approach:

Strengths

- There is a strong rebuttal to the criticism that follow-up of these cohorts is relatively brief—only 3 years or so. First, when it comes to analysis of novel data, one must start somewhere; and second there is some suggestion that more advanced, psychometrically matched summary measures of the burst data might be extra sensitive to relatively short-term decline.
- The criticism that the donor cohorts are not representative population-based samples is not appropriate. Such an argument would effectively nullify the entire ADRC / ADCC system in the US. It is entirely natural to collect this kind of novel data in relatively small convenience samples of hundreds of older adults at first, then push the method out to larger and more representative samples later. In addition, as noted, the EAS and ESCAPE recruited from voting rolls and thus represent the local population.

- The relative lack of biomarkers in the prior submission was addressed by introducing the relatively cheap and easy to ascertain cardiometabolic / inflammatory markers into the analytic plan, rather than expensive amyloid and tau measurements. This approach is a reasonable middle ground that pushes the project further toward biology, without making it infeasibly large.

Weaknesses

- None noted.

5. Environment:
Strengths

- The [redacted] environment seems adequate for doing this research

Weaknesses

- None noted.

Protections for Human Subjects:

Acceptable Risks and/or Adequate Protections

Data and Safety Monitoring Plan (Applicable for Clinical Trials Only):

Not Applicable (No Clinical Trials)

Inclusion of Women, Minorities and Children:

- Sex/Gender: Distribution justified scientifically
- Race/Ethnicity: Distribution justified scientifically
- For NIH-Defined Phase III trials, Plans for valid design and analysis: Not Applicable
- Inclusion/Exclusion of Children under 18: Excluding ages <18; justified scientifically
- It's a study of aging, with no neurodevelopmental angle.

Vertebrate Animals:

Not Applicable (No Vertebrate Animals)

Biohazards:

Not Applicable (No Biohazards)

Resource Sharing Plans:

Acceptable

Authentication of Key Biological and/or Chemical Resources:

Not Applicable (No Relevant Resources)

Budget and Period of Support:

Recommend as Requested

THE FOLLOWING SECTIONS WERE PREPARED BY THE SCIENTIFIC REVIEW OFFICER TO SUMMARIZE THE OUTCOME OF DISCUSSIONS OF THE REVIEW COMMITTEE, OR REVIEWERS' WRITTEN CRITIQUES, ON THE FOLLOWING ISSUES:

PROTECTION OF HUMAN SUBJECTS: ACCEPTABLE

COMMITTEE BUDGET RECOMMENDATIONS: The budget was recommended as requested.

Footnotes for [redacted]

Ad hoc or special section application percentiled against "Total CSR" base.

NIH has modified its policy regarding the receipt of resubmissions (amended applications). See Guide Notice NOT-OD-14-074 at http://grants.nih.gov/grants/guide/notice-files/NOT-OD-14-074.html. The impact/priority score is calculated after discussion of an application by averaging the overall scores (1-9) given by all voting reviewers on the committee and multiplying by 10. The criterion scores are submitted prior to the meeting by the individual reviewers assigned to an application, and are not discussed specifically at the review meeting or calculated into the overall impact score. Some applications also receive a percentile ranking. For details on the review process, see http://grants.nih.gov/grants/peer_review_process.htm#scoring.

MEETING ROSTER

The roster for this review meeting is displayed as an aggregated roster that includes reviewers from multiple CSR Special Emphasis Panels of the Aggregate Roster
for the 2019/05 council round.
This roster for CSR is available at:
[redacted]
